# Assessing, categorizing and proposing the therapeutic effect of complete dentures on temporomandibular disorders analyzed by craniomandibular index: a prospective interventional study

**DOI:** 10.1186/s12903-025-05979-3

**Published:** 2025-04-16

**Authors:** Sharayu Nimonkar, Surekha Godbole, Vikram Belkhode, Pranali Nimonkar, Sheetal Khubchandani, Namita Zilpilwar

**Affiliations:** 1 Department of Prosthodontics, Sharad Pawar Dental College & Hospital, Datta Meghe Institute of Higher Education and Research, (Deemed to be University, Sawangi (Meghe), Wardha, Maharashtra India; 2 Prosthodontist, Wardha, Maharashtra India; 3 Reader, Trauma Care Centre, Government Medical College, Nagpur, Maharashtra India; 4 Perusing MDS Prosthodontics, Sharad Pawar Dental College & Hospital, Datta Meghe Institute of Higher Education and Research (Deemed to be University, Sawangi (Meghe), Wardha, Maharashtra India; 5 Prosthodontics, Fellow Maxillofacial Prosthodontics, New SBI Colony Nisarg Nagri, Nagpur Road, Wardha, 442001 India

**Keywords:** Complete denture prosthesis, Edentulous, Helkimos index, Temporomandibular disorders

## Abstract

**Background:**

Edentulism is a devastating condition that is often irreversible and considered a marker of a diseased state of oral health. The prevalence of edentulism globally is 22%, which is quite high. The long-term absence of complete dentures is thought to cause joint problems.

**Objectives:**

To evaluate and categorize the effect of complete dentures on signs and symptoms of temporomandibular disorders (TMD) analyzed by the Craniomandibular Index (CMI) in completely edentulous patients.

**Methods:**

This interventional and prospective study was conducted. Preliminary screening of completely edentulous patients with the Anamnestic component of the Helkimos Index was done. 110 subjects with severe TMD, as per the preliminary screening, were included in the study. Secondary Screening for the Dysfunction and Palpation components of the CMI was done to obtain baseline measures. The complete denture was delivered. After three months, the CMI Score was re-evaluated. Descriptive and analytical statistics were done.

**Results:**

The mean CMI score before denture insertion was 1.50 ± 0.12, and after denture insertion, it was 0.45 ± 0.25. The test used for the analysis of the values was the student’s paired t-test. A statistically significant difference in CMI at pre- and post-treatment with complete dentures (t = 57.90, *p* = 0.0001) was found.

**Conclusion:**

The study results showed abate in the severity of the signs and symptoms of TMD among the complete denture wearers over 3 months. The result of current study established the remedial effect of complete dentures on TMD and also gave a new parameter to contemplate the line of treatment by categorizing the severity of TMD on the basis of the score obtained.

## Introduction

A wide range of issues about the muscles of mastication, occlusion, and the temporomandibular joint (TMJ) are together referred to as “temporomandibular disorder” (TMD) [1]. These are featured as sound/click in the joint area or a feeling of fatigue, stiffness during mouth opening or upon waking up, pain in the masticatory muscles or TMJ area, mandibular luxation or locking during mouth opening, and discomfort during mouth opening [2].

Several published studies quoted that TMD is remarkably common in the general population [3]. The prevalence of TMD in adults over 65 years of age is high at 3–5% of the US population [4]. Back K et al. showed approximately 45–70% of people over the age of 65 with evidence of TMD prevalence [5]. TMD was present in 36.99% of dental students of Saudi Arabia as per Srivastava KC et al. [6].

The etiology of TMD has been a contentious area of debate [7]. Originally, occlusal differences were considered the main factor behind TMD [8]. However, emotional trauma and occlusal disparity were suggested as potential causes by some authors [9]. As further research was conducted on TMD patients, it became evident that the etiology could involve psychosocial, psychological, and physical components [10]. TMD is commonly treated with the care of the personal being, physical therapy, splints, physiotherapy, behavioral therapy, relaxation methods, products or medicines that relax muscles, and pharmaceutical therapies [11].

The documented literature states that symptoms of Temporomandibular Joint Dysfunction are not as severe in edentulous people as they are in those with natural dentition, as the proprioceptive response from teeth is no longer present to trigger the symptom of TMD [12]. The above argument is debatable in those who have been edentulous for a long time and have not worn dentures. The alterations in the vertical and horizontal positions of the mandible, along with changes in the location of the condyles within the mandibular fossae are thought to predispose to TMD [13].

Diagnosing the causes of pain and dysfunction related to TMD is important to guide treatment decisions. The best method of diagnosing TMD is clinical examination. Imaging is considered to be a useful adjunct method. Questionnaire-based methods or indexes are subjective and range the pain severity associated with joints and muscles [14]. There are no criteria for determining the severity of TMD with a number value except indexes. The literature describes several instruments for TMD diagnosis, but no consensus diagnostic criterion has been established [15]. Indexes were further modified by adding the clinical examination components along with the questionnaire, increasing their weightage in diagnosing TMD.

More specific diagnoses are usually required if the management progresses beyond conservative options. The craniomandibular index (CMI) includes both subjective and objective assessments [16]. The current study focused on assessing conservative treatment offered to the completely edentulous population in the form of complete dentures and hence CMI was used as a diagnosing modality.

Currently, the awareness regarding immediate rehabilitation post-edentulism is less, which is the major reason for the development of TMD in edentulous subjects. The signs of TMD in edentulous subjects are often ignored and misunderstood. And hence the study was designed to evaluate the changes in the severity of the signs and symptoms of TMDs before and after prosthetic rehabilitation of completely edentulous patients with complete dentures. And based on the score obtained categorization of severity is done and treatment is formulated for each category.

## Methodology

A Prospective interventional study was carried out. Random selection of completely edentulous subject with severe TMD reporting to the outpatient department, who needed dental treatment was done. The duration of the study ranged from December 2019 to December 2022.

### Ethical aspects

The approval for the study was attained from the Institutional Ethical Committee (IEC/2018-19/7344). After approval, the study participants were enrolled. Before initiating the process of complete denture fabrication, the written consent of all the patients participating in the study was obtained.

### Calculating the sample Size-

N Master V.2.0 was the program used to calculate the sample size.

$$\:\text{n}\:=\:\frac{2{\upalpha\:}\:/2\:2\:.\:\text{P}.\:(1-\text{P})}{\text{d}2}$$

Where,

2 α /2 = level of significance at 5% i.e. 95%.

confidence interval = 1.96.

*P* = prevalence of signs and symptoms.

= 15% =0.15

D = error of margin = 7% =0.07.

n = *1.962 × 0.15 × (1-0.15)*.

0.072

= 99.96

= 100

Assuming a 10% non-response rate, the Sample size will be, 100 + 10 = 110.

110 patients were included in the study.

### Study population

#### Inclusion criteria

1. The subject should be healthy.
2. Subjects from both genders.
3. Age group ranging between 40 and 70 years.
4. Subjects whose last tooth in the mouth was extracted between the period of 6 months to 5 years. (Confirmed on history and documentations if available)
5. Subjects with severe TMD as per the scale of anamnestic component of Helkimo’s Index.

#### Exclusion criteria

1. Subjects that do not cooperate.
2. Subjects who wear complete dentures.
3. Subjects with previously diagnosed TMD and who underwent treatment for the symptoms.

## Method

### Step 1- preliminary screening with anamnestic component of Helkimo’s index

Utilizing the anamnestic component of the Helkimos Index, a preliminary screening was conducted to select study participants. The anamnestic component is a survey using a “Yes or No” format for responses to a questionnaire. The signs and symptoms of TMD were identified by exposing the completely edentulous patients to a questionnaire [17]. The responses of the patients were analyzed and graded.

### Step 2- secondary screening

**Fig. 1:**
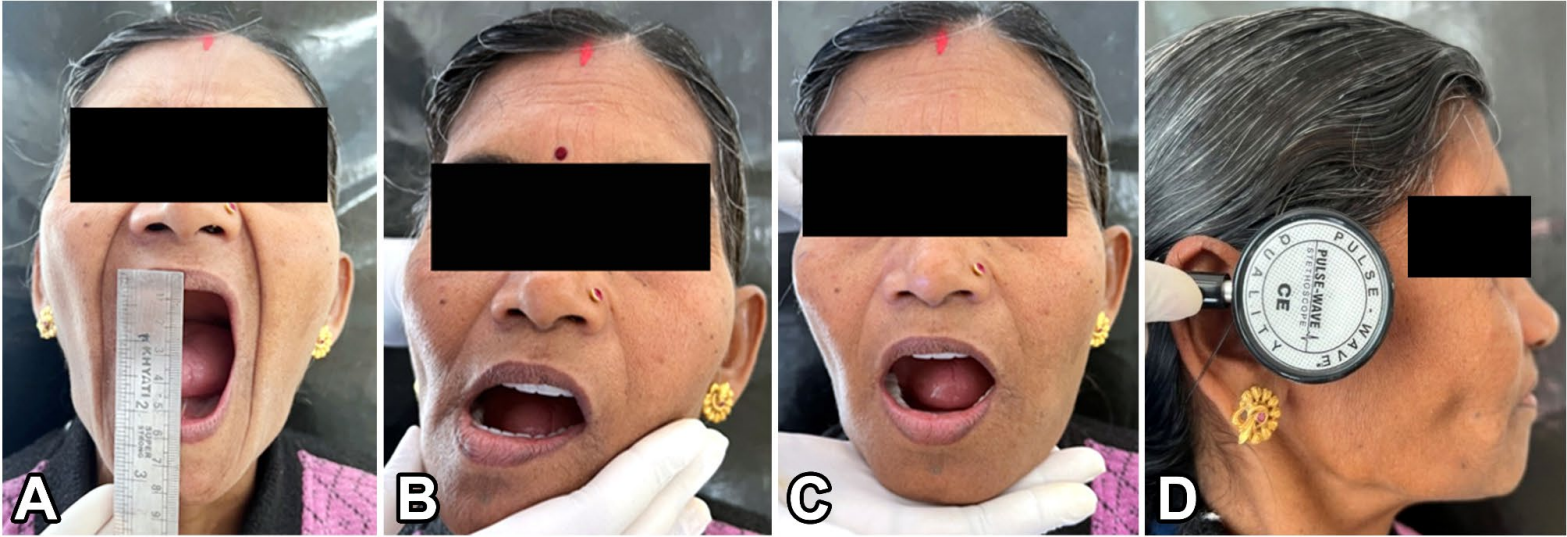
Evaluating Dysfunction Index of CMI: **A** Maximum opening. **B** Deviation on Opening or closing. **C** Restriction on opening. **D** TMJ Noise

**Fig. 2:**
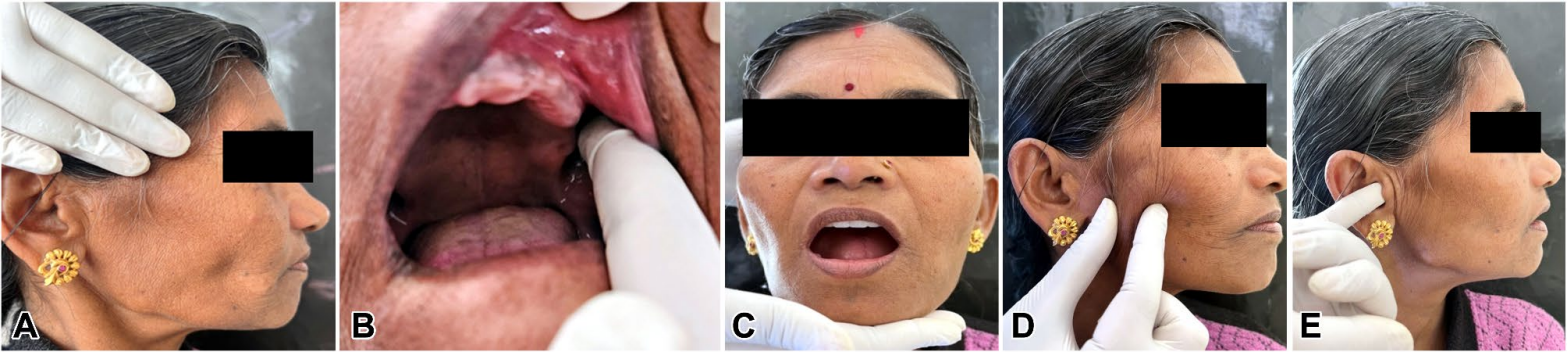
Evaluating Palpation Index of CMI: **A** Palpation of temporalis. **B** Palpation of temporalis muscle to elicit tenderness. **C** Palpation of lateral pterygoid muscle. **D** Palpation of masseter muscle. **E** Palpation of the joint

Following screening, the patients who exhibited severe symptoms were chosen to participate in the study and had additional screening under the Dysfunction Index (DI) and Palpation Index (PI) components of the CMI to obtain baseline measures (Figs. 1 and 2). The CMI score was calculated as per the formula given by Friction J et al. [16]. Three months after the denture was inserted, the CMI score was again recorded.

### Step 4- intervention with complete denture

Under the guidance of two subject experts, a single operator carefully followed all clinical and laboratory procedures for every subject using standard tools and supplies. Heat-cure acrylic resin was used to create each denture (DPI).

### Statistical analysis

Data was collected, arranged in tabular form, and analyzed using Statistical Package for the Social Sciences (SPSS) software Version 24.0 (IBM Corporación, Chicago, USA). Statistics were done by analytical and descriptive type. The data is represented in numbers, percentages, means, and standard deviations. Shapiro-Wilk test was used to analyze the normality of the data which is continuous. Also, a parametric test (t-test) was used as the data followed a normal distribution. The independent sample t-test was used to compare mean differences between groups. The *p*-value < 0.05 is statistically significant.

## Discussion

The TMD and tooth loss are directly related. Entirely edentulous people have incredibly high rates of both TMD prevalence and severity [18]. It has been documented that differences in various angles brought on by tooth loss impact the TMJ’s mechanics, leading to TMD [19]. The long-term absence of a complete denture prosthesis causes the mandible to migrate from its centric relation to a habitual centric posture because of the loss of vertical dimensions. Similarly, for those who are edentulous, psychological issues brought on by tooth loss, aging, and emotional stress all play a significant role in the development of TMD [20].

The responses from all the 110 edentulous study subjects were recorded. The mean DI score at pretreatment was 0.82 ± 0.11, and at post-treatment, it was 0.22 ± 0.05. The mean PI score at pretreatment was 1.66 ± 0.30, and at post-treatment, it was 0.53 ± 0.38. Using the Student’s paired t-test, a statistically significant difference was seen in both DI and PI at pre- and post-treatment (t = 26.94, *p* = 0.0001). As per the statistical values, before intervention with a complete denture, the score of CMI was 1.50 ± 0.12, and after 3 months, it decreased to 0.45 ± 0.25, which suggests that there was a lowering of the number of subjective signs and symptoms of TMD after denture use [Table 1]. The comparison of individual parameters of DI, revealed that only clinically can lock open (subluxate) showed no change after treatment also, it remained as it was even after complete denture delivery. All the other parameters showed a statistically significant reduction in the value after denture insertion. The comparison of individual parameters of PI showed that TMJ noise had a drastic fall with a *p*-value of 0.35, NS. In the PI most promising result was found for TMJ capsules and intra-oral muscles with a *p*-value of 0.0001, S, and 0.0008, S respectively. Extra-oral muscles and neck muscle pain also showed a reduction in the score that was statistically significant. Table 2 is the post-treatment categorization of the study subject based on CMI score.

**Table 1:** Comparison of DI, PI, CMI before and after treatment student’s paired t-test

		Mean	Normal	Standard Deviation	Standard Error Mean	Mean Difference	test-value
DI	Pre-Treatment	0.82	110	0.11	0.01	0.60 ± 0.10	61.34 *P* = 0.0001, S
	Post Treatment	0.22	110	0.05	0.005		
PI	Pre-Treatment	1.66	110	0.30	0.02	1.12 ± 0.43	26.94 *P* = 0.0001, S
	Post Treatment	0.53	110	0.38	0.03		
CMI	Pre-Treatment	1.50	110	0.12	0.01	1.05 ± 0.19	57.90 *P* = 0.0001, S
	Post Treatment	0.45	110	0.25	0.02		

**Where P- probability**

**Table 2:** Categorization of the CMI score

	Normal	Mean	Standard Deviation	Standard Error	95% Confidence Interval for Mean		Min.	Max.		
					Lower Boundary	Upper Boundary			F-value	*P*-value
Mild Severe	31	1.3881	0.05147	0.00924	1.3692	1.4069	1.32	1.43	166.946	< 0.01
Moderate Severe	45	1.4756	0.02625	0.00391	1.4677	1.4834	1.44	1.50		
High Severe	34	1.6532	0.09177	0.01574	1.6212	1.6853	1.54	1.76		
Total	110	1.5058	0.12107	0.01154	1.4829	1.5287	1.32	1.76		

**Where P- probability**,** F-indicates statistics of one way ANOVA**

The present study reported a high prevalence of TMD among the edentulous subjects when screened with an anamnestic component of the Helkimos index. Shetty R. researched to determine the prevalence of TMD in a community of entirely edentulous people [21]. He said 59% of research participants had more than two TMD symptoms. In 2019, AlZarea BK also found that 60.5% of asymptomatic, fully edentulous subjects had a high prevalence of TMD [22]. Similar findings were reported by Shi and Wang et al., who discovered that 43.2% of their study participants (who were entirely edentulous) had clinically positive indications of TMD [23].

One hundred ten edentulous non-denture-wearing patients with anamnestic or clinically severe TMD signs and symptoms were included in the current investigation. The study’s findings showed statistically significant differences in the signs and symptoms of TMD among edentulous subjects before and after three months of denture insertion. Our findings suggest that the use of dentures improves and reduces the severity of TMD and has a noticeable effect on the TMJ changes among entirely edentulous patients. Among the different parameters of dysfunction index jerky opening or closing, “S” Deviation on Opening or closing, Lateral Deviation at Full Opening, Rigidity of Jaw on Manipulation, TMJ Noise, Crepitus, Popping, Protrusion–Pain, and Protrusion–Pain was the most common finding among edentulous subjects. Among the palpation index, the most common finding was with Middle temporal, Lateral pterygoid, Medial pterygoid, Superior sternocleidomastoid, and Superior capsule. Our study results are like those of Meyerowitz et al., who reported that 32% of participants had muscle pain on palpation [24].

The present study has further categorized the score obtained for patients with TMD into low, medium, and high categories. The category was made based on the score. Low had a range of scores between 1.32 and 1.43. Medium had a range of 1.44 to 1.50 and high had a range between 1.54 and 1.76. This categorization was based on score and was not subjected. The categorization helped to get the severity Index. The symptoms and treatment planning are also sorted according to category. The following symptoms and indicators of TMD were seen in the low category: limited mouth opening, jaw deviation when opening the mouth, headache, joint pain, muscle soreness, pain in the area where the cervical and lower jaw muscles meet, crepitus, and clicking sounds in the joint. These symptoms responded quickly and showed remission in their severity after being intervened with complete denture prosthetic rehabilitation (Table 3).

**Table 3:** Signs and symptoms prevalent in low category TMD along with its treatment protocol

Category with score	Signs and Symptoms showing positive changes towards remission of disease		Treatment option
Low (1.32 to 1.43)	DI	PI	A. Prosthetic rehabilitation B. Counseling The factors that aggravate pain can be avoided using counseling help and educating the patient. C. Avoid over use of jaw muscles Eat soft, cut food into small pieces. Steer clear of sticky or chewy food. Avoid chewing gum.
	Passive stretch opening	T.M.J. Capsules pain	
	Pain on opening	Temporalis muscle pain	
	Restriction on opening	Masseter Muscle pain	
	Jerky opening or closing	Lateral pterygoid	
	Lateral deviation at whole opening	Medial pterygoid	
	Protrusion–pain		
	Protrusion–limitation		
	Rigidity of jaw on manipulation		
	T.M.J. Noise		

The medium category showed muscle pain, TMJ capsule pain, popping, pain and restricted lateral movement, sub-luxation, and luxation. This category showed a lower post-treatment effect with complete dentures on the severity of TMD. It suggests the combination of the complete denture with one or two of the additional treatments from physical therapy, physiotherapy, behavioral therapy, relaxation techniques, muscle-relaxing equipment, and medicinal treatment options to be included for more effectiveness (Table 4).

**Table 4:** Signs and symptoms prevalent in medium category TMD along with its treatment protocol

Category with score	Signs and Symptoms showing positive changes towards remission of disease		Treatment option
Medium (1.44 to 1.50)	DI	PI	A. Prosthetic rehabilitation (A options mentioned for low category combined with 2 to 3 treatment modalities mentioned below} B. Physical therapy C. Physiotherapy D. Behavioural therapy E. Relaxation techniques F. Muscle-relaxing equipment (Biofeedback) G. Medicinal treatment options H. Acupuncture I. Injections [Corticosteroid or Botulinum toxin type A]
	Laterotrusion pain	T.M.J. Capsules pain	
	Laterotrusion limitation	Masseter muscle pain	
	Crepitus-Coarse	Lateral pterygoid	
	Reproducible laterotrusive click	Medial pterygoid	
	Protrusion Pain		
	Pain on opening		

The high category showed neck muscles, intraoral and extraoral muscles involved along with non-reproducible clicks and reciprocal clicks. This category of patients showed less amount of remission as compared to the other two groups. This finding suggested surgical intervention for treating TMD (Table 5).

**Table 5:** Signs and symptoms prevalent in high category TMD along with its treatment protocol

Category with score	Signs and Symptoms showing positive changes towards remission of disease	Treatment option	Category with score
High (1.54 to 1.76.)	DI	PI	Surgical approach 1. TMJ arthroscopy 2. Modified condylotomy (For pain and locking) 3. Open-joint surgery 4. Acupuncture 5. Injections [Corticosteroid or Botulinum toxin type A]
	Clinically can lock open	Posterior digastric muscle pain	
	Locked closed with condylar translation	Vertex muscle Pain	
	Rreproducible opening and closing click	Sternocleidomastoid muscle pain	
	Non reproducible opening click	Trapezius pain	
	Popping	TMJ capsule pain	

Indexes are a secure and trustworthy diagnostic and severity grading technique for TMDs [25, 26, 27]. Numerous writers have included a range of verified and certified indices in their TMD-related epidemiological research [28, 29]. The present study has used an anamnestic component of Helkimo’s index for screening. This component helped to screen the patients to get the desired samples with severe TMD. The DI and PI component of CMI was used to find scores to determine the severity of TMD. Unlike other indices, the CMI is accurate as it gives the score. Although the anamnestic component was subjective, the score obtained by the dysfunction and palpation component verified its reliability. There are currently not enough randomized clinical trials to support the efficacy of the TMD treatments being used on fully edentulous people [30]. The purpose of this study was to evaluate whether complete dentures that are precisely made can lessen the severity of TMD in those who are entirely edentulous. The grading system for evaluating the severity of TMD has been superseded by taking the score to assess the TMD to evaluate the exact amount of remission of the disease. All the grading systems documented are based on subjective analysis. This score is used as a valid parameter to categorize the severity of TMD and to establish the treatment protocol depending upon the parameters affected in the particular grades of CMI. The study also generates evidence for the type of category of CMI that can benefit from prosthetic rehabilitation of edentulous patients and parameters under each grade such as intraoral, extra-oral, muscles, neck muscles, TMJ capsules, TMJ noises, pain, restricted and deviated jaw opening, rigidity and stiffness of the joint. This will help to generate a treatment protocol for TMD among edentulous patients depending on the severity and factors affected.

The overall limitation of the study was the period of follow-up which was less. The challenge was to obtain the willingness of patients to participate in the study. There is a scope of research to find the permissible period of edentulism before the development of TMD. The future overview would be to determine the gender prediction to assess which gender is more prone to TMD after being edentulous. Longitudinal studies can be undertaken to investigate the correlation of age with the severity of TMD in the edentulous population. Studies can be undertaken using more objective methods for diagnosing severity of TMD using electro-myography and other recent artificial intelligence based applications. Perception, Knowledge, and practice of assessing and treating TMD among dentists have to be evaluated and a study targeting a massive population can be undertaken.

## Conclusion

The number of edentulous patients has steadily grown as the population’s aging process has accelerated. This study evaluated the remedial effect of complete dentures on lowering the number of subjective signs and symptoms of TMD among edentulous patients. This study’s results have given several measures and have not graded the severity of the disease, so they are more reliable than any subjective grading parameter. It has established a new categorizing system in CMI for severe TMD based on the score of the present study.

## Data Availability

The datasets used and/or analyzed during the current study are available from the corresponding author on reasonable request.

## Abbreviations

TMJ: Temporomandibular Joint
CMI: Craniomandibular Index
DI: Dysfunction Index
PI: Palpation Index
SPSS: Statistical Package for the Social Sciences
